# Two Cases of Dropsy, Dependent upon Bright’s Disease, Cured by Scoparius

**Published:** 1873-11

**Authors:** 


### TWO CASES OF DROPSY, DEPENDENT UPON BRIGHT'S DISEASE, CURED BY SCOPARIUS.

Case I. You would not suppose much to be the matter with this man, yet if you had seen him two weeks ago you would have seen a very sick man. He came into the hospital immensely swollen, about as marked a case of dropsy as could be found. Briefly, the following is his history:

Thomas S., a printer, aged 45 years, was admitted December 16th, 1872. He had a chancre six years ago, but no constitutional symptoms followed. For the last year, as a result of mental depression, he has been a hard drinker, and he has not felt well since he commenced his course of intemperance. Six months ago he noticed that his feet were much swollen at night, especially after standing at his printing case. Five weeks ago, boils came out upon his legs, and three weeks ago he was obliged to stop work on account of general dropsy. For two weeks he passed very little water. His urine was acid, s. g. 1018, and contained a heavy deposit of albumen upon applying the heat and nitric acid test. The albuminous deposit filled from one-third to one-half of the test tube. Under the microscope his urine shows fatty, hyaline casts, with occasional epithelium cells and granular matter.

This man, therefore, had Bright's disease, with general dropsy dependent upon it. Taking in to account the origin of the case, and moreover, examining into the character of the deposit in the test tube, there could be no doubt that we were dealing with a case of large kidney undergoing fatty degeneration. When you have, in a chronic case, a large amount of albumen in the urine, extensive dropsical swelling, and you find tube casts, granular and fatty, there can be no possible mistake in the diagnosis The more fatty the casts, the more fatty will be the kidney, and the less fatty the casts, the more likely the kidney with fatty degeneration about to commence. Take these facts and combine them with the man's previous condition—depressed and intemperate—and we have the true solution of his trouble.

The urgent symptioms of this man were those of dropsy. It was for this that he consulted us, and we set to work to get rid of it. Remembering the urine was scanty, we treated it more with diuretics than is our habit. Scoparius, or broom, was the one made use of. He commenced active treatment about the 20th of December, taking an ounce of broom in a pint of water daily. He continued its use until within a few days, and under it we have had a complete disappearance of the swelling. There is nothing now to show for his previous dropsy, and that you may judge how much the urinary secretion has been increased, it is only necessary to state that while previous to the administration the greatest amount in any one day was 48 ounces, since he commenced taking the infusion of broom the average daily amount is 68 ounces.

This man now wants to leave the hospital; but I think before we let him go the treatment of his remaining affection, the condition of his kidney, should be insisted upon. Tincture of the chloride of iron, taken for weeks and months, with an occasional intermission, in doses of from 20 to 30 minims, three times daily, will best meet his condition. Along with the iron he should have good food, but avoid the use of stimulants. By carrying out this plan of treatment we may keep in check the change that is now going on in the kidney. Should dropsy at any time recur, we will return to the administration of the broom.

In a case like this, hot baths and diaphoretics, like Dover's powder, will materially aid the action sought.

Case II. Here is another specimen of the same kind of trouble. This is an old case; the patient, J. D., came into the hospital July 29th, 1872. As in the preceding patient, on admission he was intensely swollen. His urine contained a heavy deposit of albumen, and under the micrsocope, showed fatty and granular casts, with here and there some hyaline casts; the s. g. varied from 1020 to 1024. We had no doubt of the character of the case. It was one of granular degeneration, with commencing fatty change and dropsy. He had first administered to him a mixture of iron and gentian. He also took nux vomica, and a mixture containing in each dose three drops of the tincture of iodine and ten drops of the aromatic spirits of ammonia. Under these varied treatments he improved somewhat but the dropsy did not diminish as it should. Then he was placed upon juniper berries, and finally took a tonic diuretic, composed largely of the tincture of chloride of iron and acetate of ammonia, a favorite prescription in this hospital. Even this did not answer the purpose. Seeing the favorable effect of scoparius, I finally put him upon the use of the same agent, and I have a gratifying result to record. He is to-day, very nearly free from dropsy. Some swelling of the limbs remains, but the ascites has almost disappeared. He has no heart murmur, no pericardiac effusion. We can do nothing better here than continue the broom, as under its use he passed more water and is more comfortable. With it we shall use thirty miniums of the tincture of chloride of iron, three times daily.—*Med. and Surg. Reporter—Clinic of Prof. J. M. DaCosta, Pennsylvania Hospital.*

